# Molecular evolution of the three short PGRPs of the malaria vectors *An*opheles *gambiae *and *Anopheles arabiensis *in East Africa

**DOI:** 10.1186/1471-2148-10-9

**Published:** 2010-01-12

**Authors:** Cristina Mendes, Rute Felix, Ana-Margarida Sousa, Joana Lamego, Derek Charlwood, Virgílio E do Rosário, João Pinto, Henrique Silveira

**Affiliations:** 1 Centro de Malária e outras Doenças Tropicais, UEI Malária, Instituto de Higiene e Medicina Tropical, Universidade Nova de Lisboa, Rua da Junqueira, 96, 1349-008 Lisbon, Portugal; 2 DBL - Centre for Health Research and Development, Faculty of Life, 57 Thorvandersvej, Fredriksberg, Copenhagen, Denmark

## Abstract

**Background:**

Immune responses to parasites, which start with pathogen recognition, play a decisive role in the control of the infection in mosquitoes. Peptidoglycan recognition proteins (PGRPs) are an important family of pattern recognition receptors that are involved in the activation of these immune reactions. Pathogen pressure can exert adaptive changes in host genes that are crucial components of the vector's defence. The aim of this study was to determine the molecular evolution of the three short PGRPs (PGRP-S1, PGRP-S2 and PGRP-S3) in the two main African malaria vectors - *Anopheles gambiae *and *Anopheles arabiensis*.

**Results:**

Genetic diversity of *An. gambiae *and *An. arabiensis *PGRP-S1, PGRP-S2 and PGRP-S3 was investigated in samples collected from Mozambique and Tanzania. PGRP-S1 diversity was lower than for PGRP-S2 and PGRP-S3. PGRP-S1 was the only gene differentiated between the two species. All the comparisons made for PGRP-S1 showed significant P-values for Fst estimates and AMOVA confirming a clear separation between species. For PGRP-S2 and PGRP-S3 genes it was not possible to group populations either by species or by geographic region. Phylogenetic networks reinforced the results obtained by the AMOVA and Fst values. The ratio of nonsynonymous substitutions (Ka)/synonymous substitutions (Ks) for the duplicate pair PGRP-S2 and PGRP-S3 was very similar and lower than 1. The 3D model of the different proteins coded by these genes showed that amino acid substitutions were concentrated at the periphery of the protein rather than at the peptidoglycan recognition site.

**Conclusions:**

PGRP-S1 is less diverse and showed higher divergence between *An. gambiae *and *An. arabiensis *regardless of geographic location. This probably relates to its location in the chromosome-X, while PGRP-S2 and PGRP-S3, located in chromosome-2L, showed signs of autosomal introgression. The two short PGRP genes located in the chromosome-2L were under purifying selection, which suggests functional constraints. Different types of selection acting on PGRP-S1 and PGRP-S2 and S3 might be related to their different function and catalytic activity.

## Background

Mosquito immune responses to *Plasmodium *play an important role in the natural control of the infection and are initiated when pathogen-associated molecular patterns (PAMPs) are recognized by pattern recognition receptor (PRR) molecules [1]. Peptidoglycan recognition proteins (PGRPs) are one family of PRR, which contain a domain very similar to bacterial amidase. The *Anopheles *PGRPs can be divided into two different classes: short and long. In *Drosophila *short PGRPs are small extracellular proteins about 200 amino acids long and 18-20 kDa that are present in the hemolymph and cuticle. They are constitutively synthesized or induced mainly in the fat-body and, to a lesser extent, also in the epidermal cells, in the gut and in the hemocytes. Long PGRPs, have long transcripts and are either intracellular or membrane-spanning proteins, expressed mainly in the hemocytes [1]. Seven PGRP genes are known in the *Anopheles gambiae *genome [2]. Of these, three genes code for the short PGRPs namely PGRP-S1, PGRP-S2 and PGRP-S3. Short-PGRP genes from *An. gambiae *differ in their structure, as PGRP-S2 and S3 have predicted amidase activity while PGRP-S1 does not. The presence of catalytic activity in *Drosophila *short-PGRPs determines its function. *Drosophila *has two noncatalytic short-PGRPs, PGRP-SA and PGRP-SD, which are involved in recognition of bacteria and activation of the Toll pathway [3,4], while PGRP-SC1/2 have catalytic activity and can specifically control the level of activation of the IMD signalling pathway [5]. Transcription alteration of short-PGRPs in response to *Plasmodium *infection [6] have been reported. However, the way these molecules are involved in the response to the parasite remains unknown. The most plausible explanation in view of recent findings [7,8] is that gut microbiota modulate mosquito response to *Plasmodium*.

PGRP genes form clusters in the genome suggesting that they may have been originated by gene duplication [9]. This phenomenon is also observed in other PRR, like TEP-1, in which gene conversion plays a determinant role on their evolution [10]. The reasons why duplications occur are diverse. Once a gene is duplicated, the new gene might degenerate into a pseudogene due to recurrent deleterious mutations. However, if the duplication is advantageous for the organism, the gene might evolve new functions ("neofuntionalization") since the first copy maintains the original function. In addition duplicated genes can also have the same function but which is partially or fully subdivided between them ("subfunctionalization").

Molecular evolution of immune related genes is determined by their interaction with pathogens. In the present study we analysed patterns of evolution in three short PGRPs of the malaria vectors *An. gambiae *and *Anopheles arabiensis*, the main malaria vectors in sub-Sahara Africa, and considered the type of selective pressure acting on them.

## Results

### Polymorphism and diversity

A total of 237 sequences were analysed, 146 from *An. gambiae *(all identified as S-form) and 91 from *An. arabiensis*. Eighty eight sequences were obtained for PGRP-S1 (genbank accession nos FJ821900 - FJ821987), 47 for PGRP-S2 (genbank accession nos FJ821988 - FJ822034) and 102 for PGRP-S3 (genbank accession nos FJ821798 - FJ821899). There were 15 *An. gambiae *and 18 *An. arabiensis *for which all three genes were successfully sequenced.

In the PGRP-S1 gene, a fragment of 1182 bp that includes the coding region (552 bp) was amplified. For this gene the nucleotide diversity (π) was higher for *An. arabiensis *from Mozambique than in the other groups especially in the 5' upstream and 3' downstream non-coding regions (0.013 and 0.023 respectively) (Table 1). Overall nucleotide diversity was lower for PGRP-S1 (0.000-0.008) when compared to PGRP-S2 (0.009-0.022) and PGRP-S3 (0.002-0.027).

**Table 1:** Intraspecific polymorphism and neutrality tests for the three *Anopheles *short PGRP genes.

		*5' upstream*			*Exon 1*														*3' downstream*									
*PGRP*	*Population*	*sequence*			*UTR*			*Translated region*				*Intron*			*Exon 2*				*sequence*			*Total*				*Neutrality*		
		*L*	*S*	*π*	*L*	*S*	*π*	*L*	*S*		*π*	*L*	*S*	*π*	*L*	*S*		*π*	*L*	*S*	*π*	*L*	*S*	*π*	*πa/πs*	*Tajima's*	*Fu & Li*	
									*s*	*n*						*s*	*n*									*D*	*D'*	*F*
	Ag_Tz		5	0.002		-	-		0	1	0.000		1	0.003		1	0	0.001		0	0.000		8	0.001	0.338	-1.03*	-2.32*	-2.51*
	Ag_Mz		1	0.000		-	-		0	0	0.000		0	0.000		0	0	0.000		0	0.000		1	0.000	-	-1.11 n/s	-1.81 n/s	-1.86 n/s
S1	Aa Tz	534	1	0.000	-	-	-	255	0	0	0.000	74	0	0.000	297	0	0	0.000	32	0	0.000	1192	1	0.000	-	-1.16 n/s	-1.45 n/s	-1.57 n/s
	Aa Mz		31	0.013		-	-		3	0	0.003		1	0.004		4	1	0.004		3	0.023		43	0.008	-	-1.04 n/s	1.19 n/s	0.65 n/s
																												
	Ag_Tz		8	0.039		19	0.021		14	3	0.014		-	-		-	-	-		9	0.027		54	0.020	0.073	2.50*	1.19 n/s	1.82***
	Ag_Mz		10	0.041		27	0.022		18	3	0.014		-	-		-	-	-		17	0.038		75	0.022	0.049	0.34 n/s	0.62 n/s	0.62 n/s
S2	Aa Tz	107	1	0.002	409	5	0.005	567	11	1	0.007	-	-	-	-	-	-	-	172	14	0.024	1255	32	0.009	0.030	-0.34 n/s	-0.39 n/s	-0.43 n/s
	Aa Mz		5	0.023		7	0.007		9	2	0.007		-	-		-	-	-		6	0.015		29	0.010	0.061	0.86 n/s	0.89 n/s	0.99/s
																												
	Ag_Tz		1	0.001		-	-		10	1	0.003		-	-		-	-	-		0	0.000		12	0.002	0.015	-1.53 n/s	-1.78 n/s	-1.99 n/s
	Ag_Mz		10	0.043		-	-		28	10	0.025		-	-		-	-	-		6	0.019		54	0.027	0.141	1.08 n/s	1.21 n/s	1.38 n/s
S3	Aa Tz	84	8	0.038	-	-	-	567	16	1	0.007	-	-	-	-	-	-	-	61	6	0.028	712	31	0.012	0.038	-0.08 n/s	0.31 n/s	0.22 n/s
	Aa Mz		10	0.014		-	-		23	9	0.010		-	-		-	-	-		5	0.019		47	0.011	0.042	-1.66 n/s	-1.97 n/s	-2.19 n/s

*, **, ***represent P < 0.05, P < 0.01, and P < 0.001, ^n/s ^not significant. Ag_Tz, *An. gambiae *from Tanzania; Ag_Mz, *An. gambiae *from Mozambique; Aa_Tz, *An. arabiensis *from Tanzania; Aa_Mz, *An. arabiensis *from Mozambique; L, length; S, segregating site n - total number of nonsynonymous changes; s - total number of synonymous changes; π, nucleotide diversity.

A total of 1255 bp was amplified in the PGRP-S2 gene, which included the coding region (567 bp). In this gene the π values varied between 0.007 and 0.014 in the coding region and between 0.002 and 0.041 in the non-coding regions (Table 1). For PGRP-S3 gene a fragment of 712 bp, that included the coding region (567 bp), was amplified. The π values varied between 0.003 and 0.025 in the coding-region and between 0.000 and 0.043 in the non-coding regions.

For the three genes the total number of segregating sites was estimated for each geographic sample (Table 1). These were very similar in PGRP-S2 and PGRP-S3 genes, and much higher than in PGRP-S1. The number of synonymous and nonsynonymous substitutions was always higher in PGRP-S2 and PGRP-S3 genes than in PGRP-S1 and for those two genes synonymous changes were always higher than nonsynonymous changes. This is reflected in the π_a_/π_s _ratios that were always below one (Table 1).

### Species divergence and population differentiation

Species divergence and population differentiation were calculated for each gene of *An. gambiae *and *An. arabiensis *from two different East-Africa locations, Tanzania and Mozambique.

For the PGRP-S1 gene all pairwise Fst estimates were significant (p < 0.05). The lowest Fst estimates were obtained in intraspecific comparisons, indicating that differentiation was higher between species than between geographic populations (Table 2). For the other two genes within and between species Fst estimates were lower but still significant in almost all comparisons made (Table 2). Exceptions were for PGRP-S2 gene between *An. gambiae*_Tanzania and *An. gambiae*_Mozambique; *An. arabiensis*_Tanzania and *An. arabiensis*_Mozambique and for PGRP-S3 gene between *An. arabiensis*_Tanzania and *An. gambiae*_Mozambique.

**Table 2:** Matrix of pairwise comparisons of *F *st for the four *Anopheles *populations studied.

*Genes*		*Gamb_Mz*	*Gamb_Tz*	*Arab_Mz*	*Arab_Tz*
PGRP-S1	Gamb_Mz	-			
	Gamb_Tz	0.196*	-		
	Arab_Mz	0.950*	0.882*	-	
	Arab_Tz	0.989*	0.997*	0.034*	-
PGRP-S2	Gamb_Mz	-			
	Gamb_Tz	0.100^NS^	-		
	Arab_Mz	0.153*	0.317*	-	
	Arab_Tz	0.153*	0.317*	-0.111^NS^	-
PGRP-S3	Gamb_Mz	-			
	Gamb_Tz	0.109*	-		
	Arab_Mz	0.161*	0.347*	-	
	Arab_Tz	0.041^NS^	0.284*	0.116*	-

^NS ^- not significant; * P < 0.05; Arab, *Anopheles arabiensis*; Gamb, *Anopheles gambiae*; Mz, Mozambique; Tz, Tanzania.

To better understand the relationship between species and geographic regions, a hierarchical analysis of molecular variance (AMOVA) was performed (Table 3). For PGRP-S2 and PGRP-S3 genes most of the variation was distributed within populations, but for PGRP-S1 the remaining variation was distributed between species. These results corroborate the previous analyses indicating that for this gene the major variation occurred between species and not between different geographic regions.

**Table 3:** Hierarchical analysis of molecular variance (AMOVA) among the *An. gambiae *and *An. arabiensis *groups.

*Source of variation*	*Hierarchical AMOVA for An. gambiae and An. arabiensis*		
	PGRP-S1	PGRP-S2	PGRP-S3
Among groups	95.23	23.53	8.88
Among populations within groups	0.45	2.68	10.28
Within populations	4.31	73.8	80.84
			
Fcs (population/group)	0.095***	0.035***	0.113***
Fst (population/total)	0.957**	0.262^NS^	0.192**
Fct (group/total)	0.952*	0.235*	0.089*

^NS^- not significant; * P < 0.05; ** P < 0.01 and *** P < 0.001

### Phylogeny

**Figure 1:**
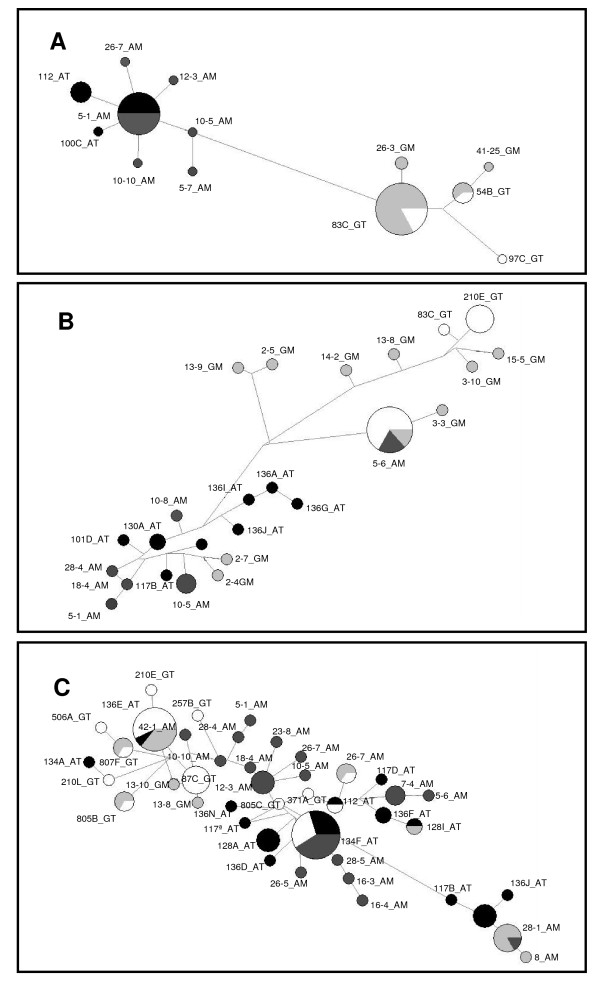
**Median-joining network for PGRP-S1, PGRP-S2 and PGRP-S3 genes**. Network was based on 13 haplotypes for PGRP-S1 gene, 25 haplotypes for PGRP-S2 gene and 43 haplotypes for PGRP-S3. The area of circles is proportional to the frequency of the haplotypes. Black - *An. arabiensis*, Tanzania; Dark-grey - *An. arabiensis*, Mozambique; Light-grey - *An. gambiae*, Mozambique and White - *An. gambiae*, Tanzania.

The median-joining network based on the PGRP-S1 haplotypes showed a clear interspecific separation (Figure 1A). Each species presented one haplotype at a higher frequency, 5-1_AM for *An. arabiensis *and 83C_GT for *An. gambiae*. Both were present in the 2 geographic regions and probably correspond to the ancestral haplotype. For *An. arabiensis *the other 7 haplotypes were unique for each geographic sample. In *An. gambiae *a lower number of haplotypes was found (5 *versus *8) and a second haplotype was shared by the *An. gambiae *- Mozambique and *An. gambiae *- Tanzania populations.

The network based on the PGRP-S2 haplotypes (Figure 1B) showed a higher number of haplotypes when compared to the PGRP-S1 network (26 *versus *13), and the separation between species was not as clear as in the network of the PGRP-S1. There was a single high frequency haplotype, 5-6_AM, that was shared by more than one population and by both species. All the other haplotypes were unique for each population and species, and showed low frequency. All 13 low-frequency haplotypes specific of *An. arabiensis *were more closely related in the network. For *An. gambiae*, 9 haplotypes grouped together in the network but two (2-7_GM, 2-4_GM) were closer to *An. arabiensis *haplotypes.

**Figure 2:**
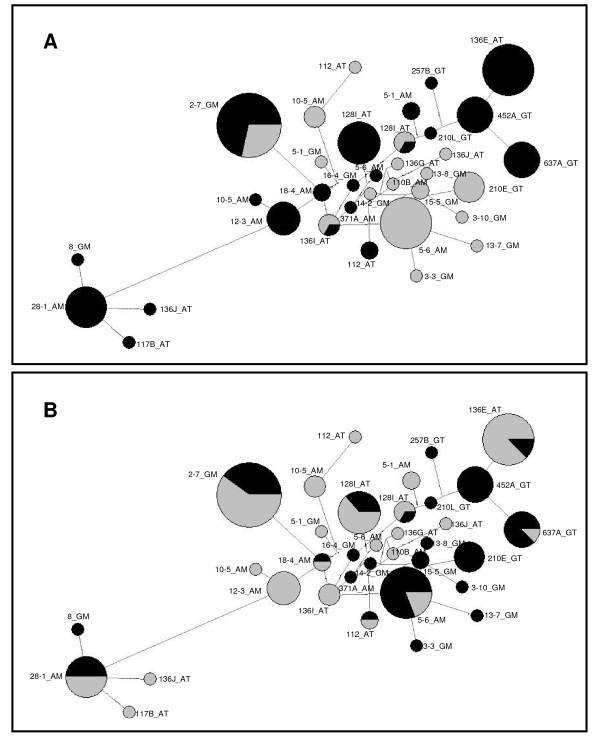
**Median-joining network for PGRP-S2 and PGRP-S3 coding regions of both *An. arabiensis *and *An. gambiae***. Network was based on thirty four haplotypes. The area of circles is proportional to the frequency of the haplotypes. A: Black - PGRP-S3, Grey - PGRP-S2; B: Black - *An. gambiae*, Grey - *An. arabiensis*.

The network based on PGRP-S3 haplotypes was the most complex. As illustrated in Figure 1C, this network presented a high number of haplotypes (N = 43). Most of the haplotypes were unique, although 5 of them were shared between species and three between geographic populations within *An. gambiae*. No clear separation was observed either between species or between geographic regions. A network based on pooled PGRP-S2 and PGRP-S3 haplotypes showed interspecific common haplotypes between these two genes (Figure 2A), namely the most frequent haplotype 2-7_GM and another 2 less frequent (136I_AT and 128I_AT). All remaining haplotypes were exclusive.

Another network based on pooled *An. gambiae *and *An. arabiensis *haplotypes (with sequences from both genes) (Figure 2B) was made to evaluate if clustering was predominately due to species or to homologous loci, in order to infer for concerted evolution. In case of concerted evolution we would expect that PGRP-S2 and PGRP-S3 would be more similar within the same species than to the homologous gene in the other species. This was not observed suggesting that gene conversion between PGRP-S2 and PGRP-S3 is not a major determinant of diversity of these genes.

### Tests for selection

For the three short PGRPs genes, neutrality tests were performed for each of the four populations separately. The Tajima's D test detected one gene, PGRP-S1, with significant departure from neutrality in the *An. gambiae*-Tanzania population. For the other genes, the results were consistent with neutral evolution (Table 1). Similarly, in Fu & Li's D and F tests only the PGRP-S1 gene showed a significant departure from neutrality for *An. gambiae-*Tanzania population. Tests for departure from neutrality can be affected by various factors such as population expansion, which would result in negative Tajima D values for all loci. However, since only one gene displayed this pattern, population expansion is thus unlikely

Synonymous (π_s_) and nonsynonymous (π_a_) nucleotide diversity was calculated for each gene within each group (Table 1) revealing values below one for PGRP-S2 and PGRP-S3, suggesting purifying selection (Table 1). A Fisher's exact test of neutrality based on the number of synonymous and nonsynonymous substitutions between sequence pairs of *An. gambiae *and *An. arabiensis *was conducted in MEGA 4.1 [11] for each collection site. P-values were equal to one for the vast majority of pairwise comparisons confirming purifying selection (data not shown). Evidence of purifying selection was also confirmed by synonymous (Ks) and nonsynonymous (Ka) divergence rates. Interspecific comparisons had Ka/Ks ratios lower than one for the three genes, particularly for PGRP-S2 and PGRP-S3, which is compatible with purifying selection (Table 4).

**Table 4:** The ratio of nonsynonymous substitutions per site (Ka) and the number of synonymous substitutions per site (Ks) in short PGRP genes between the two *Anopheles *species.

*Genes/species*		Ka/Ks*
PGRP-S1	*An. gambiae *- *An. arabiensis*	0.763
PGRP-S2	*An. gambiae *- *An. arabiensis*	0.044
PGRP-S3	*An. gambiae *- *An. arabiensis*	0.058

* Ka and Ks were estimated by DnaSP 4.0 [38] for the total coding regions

### Protein diversity

There were 14 types of proteins present in our sample [see Additional file 1: Figure S1] representing 168 individuals, six different protein sequences for PGRP-S2 and PGRP-S3 and two that were shared by both genes. *An. arabiensis *had more protein sequences at lower frequencies while the opposite was observed in *An. gambiae*. The most frequent proteins were those shared by both species (protein type 5 and 7 for PGRP-S2 and 2, 4, 9 and 10 for PGRP-S3) [see Additional file 2: Table S1].

**Figure 3:**
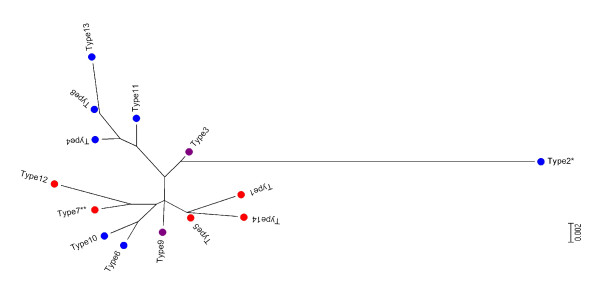
**Evolutionary relationships of the fourteen PGRP-S2/3 protein types**. The evolutionary history was inferred using the Neighbor-Joining method. The bootstrap consensus tree was inferred from 1000 replicates. Tree was drawn to scale, with branch lengths in the same units as those of the evolutionary distances used to infer the phylogenetic tree. The evolutionary distances were computed using the Poisson correction method and are in the units of the number of amino acid substitutions per site. Red dots represent PGRP-S2 proteins, blue dots represent PGRP-S3 proteins and purple represent protein types shared by PGRP-S2 and PGRP-S3.

The phylogenetic analysis using different methods (Minimum Evolution, Maximum, Neighbor-Joining and UPGMA, with or without an out group - PGRP-S1) show the same structure (data not shown). An isolated branch corresponding to PGRP-S3 type 2 was always present and was the only one with a bootstrap confidence above 70% (Figure 3).

Sequences were allocated to each protein type [see Additional file 3: Table S2] and protein types associated to haplotypes in the network (Figure 2A) revealing that the most isolated cluster of the network correspond to protein type 2 while haplotypes clustered at the opposite corner are associated with protein type 4.

**Figure 4:**
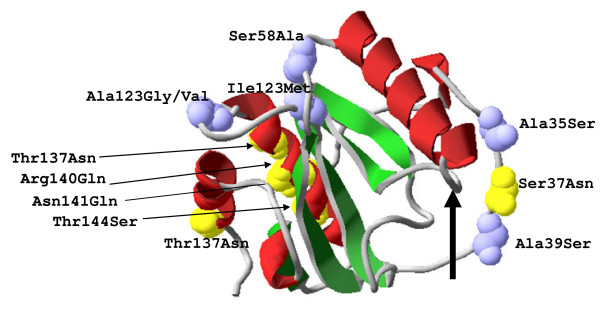
**Structural model of PGRP-S2 and PGRP-S3 proteins**. Three-dimensional (3D) structural localization of mutated amino acids represented as yellow and blue (Van de Walls spheres). The PGRP domain has three α helices (red), five β strands (green) and coils (grey); Arrow indicates the specificity-determining residues responsible for the muramyl pentapeptide - MPP-Dap recognition.

In order to understand if different protein sequences for PGRP-S2 and PGRP-S3 display different 3D configurations, proteins were modelled and visualized with the Swiss-PdB viewer v. 4.0.1. [12-14]. The best fitting 3D model for PGRP-S2 and PGRP-S3 was based on the crystal structure of the human PGRP-Iα (2aphB). The homology model shows the presence of three α helices, five β strands and coils (Figure 4).

Amino acid substitutions were mainly present at the periphery in coils. The exception was PGRP-S3 type 2 that presented 4 substitutions (Thr137Asn, Arg140Gln, Asn141 Gln, Thr144Ser) in α2 helices and 1 in α3 helices (Thr185Asn). No mutations were present in the β strands or at the recognition sites.

For PGRP-S1 there were 2 protein types, one corresponding to *An. arabiensis *and another to *An. gambiae *[see Additional file 4: Figure S2]. The 3D model showed that the amino acid substitutions between the species were also observed at the α2 helices (data not shown).

## Discussion

Nucleotide diversity estimated for the three short PGRP genes was comparable with other studies made on immune related genes of *Drosophila *and *Anopheles *[15-19]. Cohuet et al. [20] analysed 72 immune related genes of *An. gambiae *among them PGRP-S1. The nucleotide diversity (π) found for this gene for *An. gambiae *S form was 0.008; twice as high as the maximum value found in our study. This may relate to differences in geographic location. Although differences were not observed when we compared Mozambique and Tanzania a greater geographical separation could account for differences in genetic diversity between East and West African samples [20].

Some differences were found in the three genes studied: PGRP-S2 and PGRP-S3 revealed higher nucleotide diversities when compared to PGRP-S1. With respect to species divergence and population differentiation, once again PGRP-S1 differed from the others. Fst values and AMOVA indicated a clear separation between *An. gambiae *and *An. arabiensis *for PGRP-S1 gene, which was not observed for the other genes. The phylogenetic networks reinforced the results obtained by the AMOVA and Fst values. The different patterns observed between the three genes may be due to their location on the genome. PGRP-S1 gene is located in the chromosome X (within the Xag inversion) and PGRP-S2 and PGRP-S3 are located in the chromosome 2L (within the 2La inversion). *An. gambiae *and *An. arabiensis *display species-specific paracentric inversion arrangements Xag and Xbcd, respectively at the chromosome X. This should contribute to a higher differentiation between the two species at this chromosome due to reduced recombination in the case of rare hybridization between the two species. A recent study has shown that introgressed alleles between *An. arabiensis *and *An. gambiae *in the chromosome X were lost in two generations but introgressed alleles were not lost at loci located in the autosomic chromosome 2L [21]. This may explain the higher genetic divergence between species found in PGRP-S1 compared to the other two short PGRPs analysed. On the other hand, PGRP-S2 and PGRP-S3 showed no clear separation between species suggesting either retention of ancestral polymorphism or introgression between *An. gambiae *and *An. arabiensis*. Introgression of genes in autosomal chromosomes through rare hybridisation between these two sibling species has been demonstrated previously, particularly for chromosome 2L [21,22].

PRGP-S2 and PGRP-S3 are duplicated genes that are physically close (ca 3 kb) at chromosome 2L http://www.ensembl.org/Anopheles_gambiae and their coding region have 95% homology. These two genes are both considered functional, since the ratio Ka/Ks presents values much lower than 0.5 [23] and seem to be under purifying selection. This suggests subfunctionalization, i.e., these genes share the same or very similar functions. Neofunctionalization has been normally accepted has the terminal fate of duplicated genes. However more recently Gibson & Goldberg [24] suggest that this might not be a dominant mechanism of protein evolution.

This agrees with the 3D models of the protein, where substitutions were concentrated at the periphery (Figure 3), even for protein type 2 which was the most phylogenetically separated (Figure 2), while retaining the amidase/PGRP activity on both duplicate genes. Mammal PGRPs are known to form dimmers [9] and in *Drosophila *PGRP-SD interact with GNBP in order to optimally activate the Toll pathway [25]. Amino acid substitutions at non-catalytic sites that might be responsible for PRR-PRR interactions might favour the maintenance of slightly different proteins that interact optimally with different peptidoglycans. *An. gambiae *PGRP-S2 and PGRP-S3 function is not known but transcription profiles of *Plasmodium falciparum *infected mosquitoes were down regulated in midgut and carcasses when compared with mosquitoes that had a blood meal with a *P. falciparum *strain unable to produce gametocytes [6]. However, no differences were observed in both expression profiles [6]. Once again these results suggest similar function/regulation of these genes.

We cannot determine with certainty when the duplication occurred, but since it is present in both species it is likely that it happened before *An. gambiae *and *An. arabiensis *split from a common ancestor. The higher complexity of the haplotype network for PGRP-S3, showing more diversity and a higher proportion of shared haplotypes between species suggests that this may be the ancestral gene from which PGRP-S2 may have originated.

The results of selection tests showed that PGRP-S1 was the only gene presenting significant departures from neutrality with negative values for Tajima's D and Fu & Li's D and F. Negative D values indicate an excess of low frequency mutations consistent with positive selection, however this can be due to effective population size expansion. Ka/Ks ration for PGRP-S1 was also high (Table 4) but this could be a result of a very small number of diverged sites and per se do not provide a strong evidence of positive selection. For PGRP-S2 and PGRP-S3, both tests presented a non-significant deviation from neutrality. Ka/Ks ratio showed values much lower than one, reflecting functional constrains on the encoded proteins, i.e., this kind of selection contributes to the elimination of amino acid variation. Therefore, these results suggest that the PGRP-S2 and PGRP-S3 genes are under purifying selection. This does not come as a surprise as accumulating evidence suggests that the majority of *Anopheles *immune related genes, studied so far, are also under purifying selection (e.g. [26,27]). Since PGRP-S2 and PGRP-S3 could be recently duplicated genes tests like Tajima's D and Fu & Li's D and F provide limited information [28] and analysis of synonymous and nonsynonymous substitutions should be more informative. These analyses also point out to purifying selection as the driven force of short PGRPs evolution, in the same way as described for other PGRP genes of *An. gambiae *[29] or *Drosophila *[29].

The different types of selection observed between PGRP-S1 and PGRP-S2 and S3 might be a consequence of their function as PGRP-S1 does not have predicted catalityc activity and is probably involved in recognition and subsequent activation of an effector pathway, as PGRP-SA and PGRP-SD in *Drosophila *[3,4]. While PGRP-S2 and S3 would exert antimicrobial activity like PGRP-SB1 in *Drosophila *[30] and/or modulate the response as does *Drosophila *PGRP-SC1 and PGRP-SC2 [5]. Further, specificity-determining residues (Sdr) (Figure 4) will determine that distinct classes of peptidoglycans are recognized by different PGRPs: PGRP-S2 and S3 are predicted to bind MPP-Dap type peptidoglycans [31]. This does not explain why silencing of PGRP-S1 and PGRP-S2/3 protects the mosquito from infection by *Staphylococcus aureus*, that displays Lys-type peptidoglycan, but not from infection by *Escherichia coli *(Dap-type peptidoglycan) [32]. PGRP-S3 is expressed in response to gut microbiota [33]. and microbiota can modulate the response to *Plasmodium *by inducing mosquito basal immunity, which is essential to control the infection. Therefore pathogen specificity will indirectly determine the faith of mosquito malaria infection and exert different selection pressure on short PGRP coding genes.

## Conclusions

The three *Anopheles *short PGRP genes studied are involved in the recognition of pathogens. However, they show different evolutionary pathways. PGRP-S1 gene is located in the chromosome X while PGRP-S2 and PGRP-S3 are located in chromosome 2L. This explains why PGRP-S1 is less genetically diverse and shows higher divergence between *An. gambiae *and *An. arabiensis *regardless of geographic location. On the contrary, PGRP-S2 and PGRP-S3 are more diverse and less divergent due to autosomal introgression between *An. arabiensis *and *An. gambiae*. Data indicated that PGRP-S2 and PGRP-S3 are likely subject to purifying selection consistent with their role in recognising conserved PAMPs. The 3D model of the proteins showed that no mutations were present at the cleft that forms the peptidoglycan binding groove, once again implying strong evolutionary constrains probably because these proteins need to maintain their PAMP recognition site unaltered, while the periphery that interact with other molecules is more prone to accumulate variation. The lower diversity and apparently higher divergence between species for the PGRP-S2 gene suggests that this is duplication from PGRP-S3. Different types of selection acting on PGRP-S1 and PGRP-S2 and S3 might be a consequence of their different function and catalytic activity.

## Methods

### Mosquito sampling and collection methods

Samples of *An. gambiae *(82) and *An. arabiensis *(46) genomic DNA were analysed from two areas in East Africa: Mozambique and Tanzania. Mozambique samples were collected in Furvela (Inhambane province) in February/April of 2004 using light traps and samples from Tanzania were collected in Ifakara in 2000 using also light traps [34]. Mosquitoes were kept dry in single tubes with silica gel.

### Dna extraction

Genomic DNA was extracted from individual specimens according to the protocol described by Ballinger-Crabtree et al. [35]. Species identification and determination of *An. gambiae *molecular forms was carried out by PCR as described in Fanello et al. [36].

### Polymerase chain reaction and sequencing

The primers used to amplify PGRP-S1, PGRP-S2 and PGRP-S3 genes were designed based on the sequences annotated in the complete *An. gambiae s.s*. genome at Ensembl (AGAP000536; AGAP006343; AGAP006342 respectively). Name, sequence and product length of each pair of primers are represented in [see Additional file 5: Table S3].

Nested PCRs were performed in a MyCycler™ Thermal cycler (Biorad) with final reagent concentrations of 1× reaction buffer, 1.5 mM of MgCl_2_, 200 μl dNTPs, 0.5 μl of each primer and 1.25 U/μl of *Taq *DNA Polymerase (Fermentas) for all reactions. PGRP-S1 1^st ^nested PCR cycle conditions were: initial denaturation at 95°C for 2 minutes, followed by 35 cycles of 95°C for 45 seconds, 58°C for 60 seconds and 72°C for 2 minutes, with a final extension at 72°C for 5 minutes. PGRP-S1 2^nd ^nested PCR cycle conditions were: initial denaturation at 95°C for 2 minutes, followed by 35 cycles of 95°C for 45 seconds, 58°C for 30 seconds and 72°C for 60 seconds and a final extension of 72°C for 5 minutes.

PGRP-S2 and PGRP-S3 1^st ^nested PCR cycle conditions were similar to those used for PGRP-S1 amplification except for the annealing that was performed at 60°C. PGRP-S2 2^nd ^nested PCR cycle conditions were: an initial denaturation step at 94°C for 2 minutes, followed by 35 cycles of 94°C for 45 seconds, 60°C for 30 seconds and 72°C for 60 seconds, with a final step of 72°C for 5 minutes. PGRP-S3 2^nd ^nested PCR cycle conditions were similar to 1^st ^nested but the annealing temperature was 55°C.

PCR products were examined on a 2% agarose gel and products of the expected length were sequenced in both directions after purification with the SureClean Kit (Bioline) according to manufacturer's recommendations. Products were commercially sequenced by Macrogen, Korea.

### Genetic diversity and selection tests

Sequence alignments were performed using the BioEdit Sequence Alignment Editor version 7.0.5.2 [37]. Basic population genetic analyses and haplotype statistics were performed in DnaSP version 4.50.1 [38]. For each population sequence diversity was quantified by nucleotide diversity (π). To test deviation from neutrality Tajima's D test was performed. D is expected to be zero under neutrality with constant population size; Fu & Li's D and F tests were also performed; these are similar to Tajima's D test. The main differences between these tests concern the different estimators of the genetic diversity used. The Tajima's D test estimates the difference between η (total number of mutations that occurred in the entire genealogy) and π_n _(average number of nucleotide differences between two sequences) whereas in the Fu & Li's D and F tests the difference used is between the η_i _(numbers of mutations in internal branches) and η_e _(numbers of mutations in external branches) or between η_e _and π_n_.

The tests referred above give little information in the case of young genes [39]. Therefore the Ka/Ks ratio was determined which compares the number of nonsynonymous (Ka) substitutions and the number of synonymous (Ks) substitutions per site between DNA sequences. Ka and Ks ratios were estimated by DnaSP version 4.50.1 [38]. Ka/Ks ratios equal to one are expected in genes under neutrality, Ka/Ks ratios less than one indicates purifying selection and Ka/Ks ratios higher than one indicate positive selection.

### Protein diversity

Amino acid sequences were obtained using the BioEdit Sequence Alignment Editor version 7.0.5.2 [37] and aligned using the Clustal W program. Each protein sequence was modelled using swissmodel at http://swissmodel.expasy.org/workspace/index.php. The best fitting 3D model for PGRP-S2 and PGRP-S3 was based on the crystal structure of the human PGRP-Iα (2aphB) and for PGRP-S1 was based on *Drosophila *PGRP-SA (1sxrB). Nonsynonymous mutations were visualized on the models using the Swiss-PdB viewr v. 4.0.1. [14,13,12] in order to identify possible structural alterations and if location was within protein activity sites. Phylogenetic trees were constructed using MEGA 4.1 software [11].

### Genetic structure and population differentiation

The genetic structure within and among *An. gambiae *and *An. arabiensis *populations was examined with analysis of molecular variance (AMOVA) [40]. The test was performed considering each species as a distinct group. Genetic differentiation between populations was estimated by sequence-based *F *statistics (Fst) according to Hudson et al. [41]. Significance of Fst estimates was assessed by pairwise genetic distances. These tests were performed in Arlequin software version 3.11 [42].

### Phylogenetic analysis

To better understand phylogenetic relationships between intraspecific data, which normally consist in very similar sequences, we connected haplotypes on a median-joining network [43] using NETWORK 4.5.0.0 program based on default parameters.

## Authors' contributions

CM, RF, MAS and JL performed the experiments; CM, RF, JP and HS analyzed and interpreted the data; JP, JDC and VER contributed with samples. CM, RF, JP and HS wrote the paper. HS conceived and designed the experiments. All authors read and approved the final manuscript.

## Supplementary Material

Additional file 1 **Figure S1**. Multiple alignment of deduced amino acid sequences of An. gambiae and An. arabiensis PGRP-S2 and PGRP-S3. Click here for file

Additional file 2 **Table S1**. Frequencies of PGRP-S2 and PGRP-S3 protein types of An. arabiensis and An. gambiae from Mozambique and Tanzania. Click here for file

Additional file 3 **Table S2**. DNA sequences grouped by protein type. Click here for file

Additional file 4 **Figure S2**. Multiple alignment of deduced amino acid sequence of An. gambiae and An. arabiensis PGRP-S1. Click here for file

Additional file 5 **Table S3**. Sequences of primers used to amplify the three Anopheles short PGRP genes. Click here for file
