# Supplementary material for: Molecular evolution of the three short PGRPs of the malaria vectors Anopheles gambiae and Anopheles arabiensis in East Africa
Source: BMC Evol Biol. 2010 Jan 12;10:9. doi: 10.1186/1471-2148-10-9 (PMC2820002; doi:10.1186/1471-2148-10-9)
Supplement: Additional file 1 — Figure S1. Multiple alignment of deduced amino acid sequences of An. gambiae and An. arabiensis PGRP-S2 and PGRP-S3. [file 1471-2148-10-9-S1.PDF]

Additional file 1 – Figure S1 - Multiple alignment of deduced amino acid sequences of *An. gambiae* and *An. arabiensis* PGRP-S2 and PGRP-S3.

Conserved residues are marked as dots. *AGAP006342-PA; **AGAP006343-PA

```
                  10         20         30         40         50         60         70         80         90        100
         ....|....| ....|....| ....|....| ....|....| ....|....| ....|....| ....|....| ....|....| ....|....| ....|....|
Type1    MNKFATVLVL ASICLAGISA QCPRIVTRAQ WGARAASTAQ LPIRPAPWVV MHHTAGASCT TDAACAQQMR NIQSFHMDGN GWADIGYNFL VGENGAAYEG
Type2*   .......... ......V... .......... ......N... .......... .......A.. .......... .......... .......... ..........
Type3    .......... ......V... .......... .......... .......... .......A.. .......... .......... .......... ..........
Type4    .......... ......V... .......... .......... .......... .......A.. .......... .......... .......... ..........
Type5    .......... ......V... .......... .......... .......... .......... .......... .......... .......... ..........
Type6    .......... ......V... .......... .......... .......... .......... .......... .......... .......... ..........
Type7**  .......... ......V... .......... ........S. .......... .......... .......... .......... .......... ..........
Type8    .......... .....SV... .......... .......... .......... .......A.. .......... .......... .......... ..........
Type9    .......... .....SV... .......... .......... .......... .......... .......... .......... .......... ..........
Type10   .......... .....SV... .......... .......... .......... .......... .......... .......... .......... ..........
Type11   .......... .....SV... .......... .......... .......... .......... .......... .......... .......... ..........
Type12   .......... .....SV... .......... ........S. .......... .......... .......... .......... .......... ..........
Type13   .......... .....SV... .......... ....S..... .......... .......A.. .......... .......... .......... ..........
Type14   .......... V.....V... .......... .......... .......... .......... .......... .......... .......... ..........

                 110        120        130        140        150        160        170        180        190
         ....|....| ....|....| ....|....| ....|....| ....|....| ....|....| ....|....| ....|....| ....|...
Type1    RGWGRQGAHA PGYNDRSVGM GVIGTFTNAI PNAAARTAAR NLITCGVSLG HIASNYWLIG HRQAVATACP GNAFFNEIRT WPRFNPNV
Type2*   .......... .......... ........G. ......N..Q Q..S...... .......... .......... .........N ........
Type3    .......... .......... .......... .......... .......... .......... .......... .......... ........
Type4    .......... .......... ..M....... .......... .......... .......... .......... .......... ........
Type5    .......... .......... .......... .......... .......... .......... .......... .......... ........
Type6    .......... .......... ........V. .......... .......... .......... .......... .......... ........
Type7**  .......... .......... .......... .......... .......... .......... .......... .......... ........
Type8    .......... .......... ..M....... .......... .......... .......... .......... .......... ........
Type9    .......... .......... .......... .......... .......... .......... .......... .......... ........
Type10   .......... .......... ........V. .......... .......... .......... .......... .......... ........
Type11   .......... .......... ..M....... .......... .......... .......... .......... .......... ........
Type12   .......... .......... ........G. .......... .......... .......... .......... .......... ........
Type13   .......... .......... ..M....... .......... .......... .......... .......... .......... ........
Type14   .......... .......... .......... .......... .......... .......... .......... .......... ........
```
