# Supplementary material for: Molecular evolution of the three short PGRPs of the malaria vectors Anopheles gambiae and Anopheles arabiensis in East Africa
Source: BMC Evol Biol. 2010 Jan 12;10:9. doi: 10.1186/1471-2148-10-9 (PMC2820002; doi:10.1186/1471-2148-10-9)
Supplement: Additional file 2 — Table S1. Frequencies of PGRP-S2 and PGRP-S3 protein types of An. arabiensis and An. gambiae from Mozambique and Tanzania. [file 1471-2148-10-9-S2.PDF]

Additional file 2 – Table S1 - Frequencies of PGRP-S2 and PGRP-S3 protein types of *An. arabiensis* and *An. gambiae* from Mozambique and Tanzania.

Type	PGRP-S2		PGRP-S3	
	An. arabiensis N (19)	*An. gambiae* N (28)	*An. arabiensis* N (44)	*An. gambiae* N (58)
1	10.5	----	----	----
2*	----	----	11.4	8.6
3	----	7.1	2.3	----
4	----	----	2.3	50.0
5	26.3	53.6	----	----
6	----	----	----	5.2
7**	21.1	17.9	----	----
8	----	----	2.3	1.7
9	5.3	----	43.2	25.9
10	----	----	20.5	1.7
11	----	----	2.3	----
12	----	21.4	----	----
13	----	----	4.5	----
14	21.1	----	----	----
unsolved***	15.8	----	11.4	6.9

*AGAP006342-PA; **AGAP006343-PA; ***sequences with ambiguous nucleotides
