# Supplementary material for: Molecular evolution of the three short PGRPs of the malaria vectors Anopheles gambiae and Anopheles arabiensis in East Africa
Source: BMC Evol Biol. 2010 Jan 12;10:9. doi: 10.1186/1471-2148-10-9 (PMC2820002; doi:10.1186/1471-2148-10-9)
Supplement: Additional file 3 — Table S2. DNA sequences grouped by protein type. [file 1471-2148-10-9-S3.PDF]

Additional file 3 – Table S2 – DNA sequences grouped by protein type.

	PGRP-S2				PGRP-S3			
	An. arabiensis		*An. gambiae*		*An. arabiensis*		*An. gambiae*	
Protein type	Sequence name	Accession no.	Sequence name	Accession no.	Sequence name	Accession no.	Sequence name	Accession no.
1	18-4_AM	FJ822003	---	---	---	---	---	---
1	5-1_AM	FJ822007	---	---	---	---	---	---
2	---	---	---	---	28-1_AM	FJ821836	1-10_GM	FJ821801
2	---	---	---	---	101D_AT	FJ821847	14-10_GM	FJ821810
2	---	---	---	---	136G_AT	FJ821864	14-5_GM	FJ821813
2	---	---	---	---	136J_AT	FJ821866	14-8_GM	FJ821815
2	---	---	---	---	136M_AT	FJ821867	8_GM	FJ821845
3	---	---	13-9_GM	FJ821989	134A_AT	FJ821857	---	---
3	---	---	2-5_GM	FJ821995	---	---	---	---
4	---	---	---	---	43-1_AM	FJ821841	---	---
4	---	---	---	---	---	---	13-10_GM	FJ821806
4	---	---	---	---	---	---	13-7_GM	FJ821807
4	---	---	---	---	---	---	13-8_GM	FJ821808
4	---	---	---	---	---	---	13-9_GM	FJ821809
4	---	---	---	---	---	---	14-7_GM	FJ821814
4	---	---	---	---	---	---	15-3_GM	FJ821818
4	---	---	---	---	---	---	16-5_GM	FJ821823
4	---	---	---	---	---	---	16-7_GM	FJ821824
4	---	---	---	---	---	---	2-5_GM	FJ821830
4	---	---	---	---	---	---	2-8_GM	FJ821835
4	---	---	---	---	---	---	3-3_GM	FJ821840
4	---	---	---	---	---	---	136E_GT	FJ821862
4	---	---	---	---	---	---	209A_GT	FJ821871
4	---	---	---	---	---	---	210B_GT	FJ821872
4	---	---	---	---	---	---	210E_GT	FJ821873
4	---	---	---	---	---	---	210L_GT	FJ821875
4	---	---	---	---	---	---	230A_GT	FJ821876
4	---	---	---	---	---	---	352C_GT	FJ821881
4	---	---	---	---	---	---	441B_GT	FJ821883
4	---	---	---	---	---	---	452A_GT	FJ821884
4	---	---	---	---	---	---	506A_GT	FJ821886
4	---	---	---	---	---	---	54B_GT	FJ821887
4	---	---	---	---	---	---	637A_GT	FJ821888
4	---	---	---	---	---	---	783A_GT	FJ821890
4	---	---	---	---	---	---	805A_GT	FJ821891
4	---	---	---	---	---	---	805B_GT	FJ821892
4	---	---	---	---	---	---	807F_GT	FJ821895
4	---	---	---	---	---	---	807K_GT	FJ821896
4	---	---	---	---	---	---	91B_GT	FJ821898
5	26-5_AM	FJ822004	14-2_GM	FJ821990	---	---	---	---
5	43-1_AM	FJ822006	14-7_GM	FJ821991	---	---	---	---
5	5-6_AM	FJ822008	16-5_GM	FJ821993	---	---	---	---
5	101D_AT	FJ822026	2-7_GM	FJ821996	---	---	---	---
5	136G_AT	FJ822032	3-3_GM	FJ821998	---	---	---	---
5	---	---	209A_GT	FJ822009	---	---	---	---
5	---	---	210F_GT	FJ822013	---	---	---	---
5	---	---	230A_GT	FJ822015	---	---	---	---
5	---	---	257A_GT	FJ822016	---	---	---	---
5	---	---	257B_GT	FJ822017	---	---	---	---
5	---	---	441B_GT	FJ822020	---	---	---	---
5	---	---	54B_GT	FJ822010	---	---	---	---
5	---	---	772A_GT	FJ822022	---	---	---	---
5	---	---	783A_GT	FJ822023	---	---	---	---
5	---	---	807F_GT	FJ822025	---	---	---	---
6	---	---	---	---	---	---	14-2_GM	FJ821811
6	---	---	---	---	---	---	16-9_GM	FJ821825
6	---	---	---	---	---	---	805D_GT	FJ821894
7	10-10_AM	FJ821999	13-8_GM	FJ821988	---	---	---	---
7	10-5_AM	FJ822000	14-8_GM	FJ821992	---	---	---	---
7	12-10_AM	FJ822002	2-4_GM	FJ821994	---	---	---	---
7	117B_AT	FJ822028	3-10_GM	FJ821997	---	---	---	---

7	---	---	83C_GT	FJ822011	---	---	---	---
8	---	---	---	---	10-10_AM	FJ821798	257B_GT	FJ821879
9	136I_AT	FJ822033	---	---	12-10_AM	FJ821802	1-3_GM	FJ821805
9	---	---	---	---	12-3_AM	FJ821803	14-4_GM	FJ821812
9	---	---	---	---	12-6_AM	FJ821804	1-5_GM	FJ821816
9	---	---	---	---	15-2_AM	FJ821817	15-9_GM	FJ821820
9	---	---	---	---	16-3_AM	FJ821821	1-9_GM	FJ821827
9	---	---	---	---	16-4_AM	FJ821822	2-4_GM	FJ821829
9	---	---	---	---	18-4_AM	FJ821826	3-10_GM	FJ821839
9	---	---	---	---	23-8_AM	FJ821828	134F_GT	FJ821859
9	---	---	---	---	28-5_AM	FJ821838	210F_GT	FJ821874
9	---	---	---	---	100C_AT	FJ821846	256A_GT	FJ821877
9	---	---	---	---	110_AT	FJ821848	281A_GT	FJ821880
9	---	---	---	---	112_AT	FJ821849	371A_GT	FJ821882
9	---	---	---	---	117A_AT	FJ821850	772A_GT	FJ821889
9	---	---	---	---	130A_AT	FJ821855	805C_GT	FJ821893
9	---	---	---	---	133C_AT	FJ821856	83C_GT	FJ821897
9	---	---	---	---	136A_AT	FJ821860	---	---
9	---	---	---	---	136N_AT	FJ821868	---	---
9	---	---	---	---	138_AT	FJ821869	---	---
9	---	---	---	---	140A_AT	FJ821870	---	---
10	---	---	---	---	10-8_AM	FJ821800	2-7_GM	FJ821833
10	---	---	---	---	26-5_AM	FJ821831	---	---
10	---	---	---	---	27-8_AM	FJ821834	---	---
10	---	---	---	---	5-6_AM	FJ821843	---	---
10	---	---	---	---	7-4_AM	FJ821844	---	---
10	---	---	---	---	117D_AT	FJ821852	---	---
10	---	---	---	---	128I_AT	FJ821854	---	---
10	---	---	---	---	136D_AT	FJ821861	---	---
10	---	---	---	---	136F_AT	FJ821863	---	---
	---	---	---	---				
11	---	---	---	---	10-5_AM	FJ821799	---	---
12	---	---	210E_GT	FJ822012	---	---	---	---
12	---	---	210L_GT	FJ822014	---	---	---	---
12	---	---	371A_GT	FJ822018	---	---	---	---
12	---	---	403A_GT	FJ822019	---	---	---	---
12	---	---	506A_GT	FJ822021	---	---	---	---
12	---	---	804A_GT	FJ822024	---	---	---	---
13	---	---	---	---	28-4_AM	FJ821837	---	---
13	---	---	---	---	5-1_AM	FJ821842	---	---
	---	---	---	---	---	---	---	---
14	10-8_AM	FJ822001	---	---	---	---	---	---
14	130A_AT	FJ822029	---	---	---	---	---	---
14	133C_AT	FJ822030	---	---	---	---	---	---
14	136J_AT	FJ822034	---	---	---	---	---	---
Unsolved	28-4_AM	FJ822005	---	---	26-7_AM	FJ821832	15-8_GM	FJ821819
Unsolved	112_AT	FJ822027	---	---	117B_AT	FJ821851	256C_GT	FJ821878
Unsolved	136A_AT	FJ822031	---	---	128A_AT	FJ821853	454B_GT	FJ821885
Unsolved	---	---	---	---	134B_AT	FJ821858	97C_GT	FJ821899
Unsolved	---	---	---	---	136I_AT	FJ821865	---	---
