# Supplementary material for: Molecular evolution of the three short PGRPs of the malaria vectors Anopheles gambiae and Anopheles arabiensis in East Africa
Source: BMC Evol Biol. 2010 Jan 12;10:9. doi: 10.1186/1471-2148-10-9 (PMC2820002; doi:10.1186/1471-2148-10-9)
Supplement: Additional file 4 — Figure S2. Multiple alignment of deduced amino acid sequence of An. gambiae and An. arabiensis PGRP-S1. [file 1471-2148-10-9-S4.PDF]

Additional file 4 – Figure S2 - Multiple alignment of deduced amino acid sequence of An. gambiae and An. arabiensis PGRP-S1.

```
10         20         30         40         50         60         70         80         90         100

                ....|....| ....|....| ....|....| ....|....| ....|....| ....|....| ....|....| ....|....| ....|....| ....|....|
An. gambiae     LLHILVARAQ DEPAQESACP AIVKRAAWGA AKSKNVTYQL KPVANVIVHH TTGERCATVA TCKEMVANIQ TYHQTDNRWS DIGYNFLISG QNVYEGIGWH
An. arabiensis  ..L....... ...T...... .......... .......... .......... .......... .......... .......... .......... ..........

                        110        120        130        140        150        160        170        180
                ....|....| ....|....| ....|....| ....|....| ....|....| ....|....| ....|....| ....|....| ...
An. gambiae     RMGAHLRGYN DKSIGVAFLG NFDQERPTPR SLNLLARLLQ CGVELGELAD DYRLYGARQL QSTNSPGRYL YAKLQELDHW QAQ
An. arabiensis  .......... .......... ........A. .......... .......... .......... .......... .......... H..
```
