# Supplementary material for: Molecular evolution of the three short PGRPs of the malaria vectors Anopheles gambiae and Anopheles arabiensis in East Africa
Source: BMC Evol Biol. 2010 Jan 12;10:9. doi: 10.1186/1471-2148-10-9 (PMC2820002; doi:10.1186/1471-2148-10-9)
Supplement: Additional file 5 — Table S3. Sequences of primers used to amplify the three Anopheles short PGRP genes. [file 1471-2148-10-9-S5.PDF]

Additional file 5 – Table S3 - Sequences of primers used to amplify the three Anopheles short PGRP genes.

		Identification	Sequence	Product(bp)
S1	1ºnested	PGRP-S1_1st_F	5’ ACGCGATTGGTAGTGGGGTGAC 3’	1657
		PGRP-S1_1st_R	5’ ACGCGAACGAACGGGACAGGAC 3’	
	2ºnested_5’	PGRP-S1_2nd_5F	5’ TTCGCACCTACACAAACGCAGAC 3’	813
		PGRP-S1_2nd_5R	5’ CCCAGGCCGCCCGCTTCAC 3’	
	2ºnested_ 3’	PGRP-S1_2nd_3F	5’ CTCCCGTCGTCTCTTATGGTAGTA 3’	809
		PGRP-S1_2nd_3R	5’ GAACGGCAAAGAAACGGTCACAAC 3’	
S2	1ºnested	PGRP-S2_1st_F	5’ CACCAACTACGTTCCGTTGA 3’	1618
		PGRP-S2_1st_R	5’ TGTCGTCTTCCACTTCAAACA 3’	
	2ºnested_5’	PGRP-S2_2nd_5F	5’ TCACCTGTCACAATGGTCGT 3’	835
		PGRP-S2_2nd_5R	5’ CCCTGGTAGTAGCCACCGTA 3’	
	2ºnested_ 3’	PGRP-S2_2nd_3F	5’ CTCACCGACCAGGAAGTTGT 3’	835
		PGRP-S2_2nd_3R	5’ CATGTAGGCACTCACCATCG	
S3	1ºnested	PGRP-S3 new_Fwd	5’ TATGCAGCGTGACGTAGTATGG 3’	959
		PGRP-S3 new_Rev	5’ TGATTAGCACAACGATGAGATTAGC 3’	
	2ºnested	PGRP-S3 old_Fwd	5’ GGTGACTCCCCGATAACGAATAAT 3’	760
		PGRP-S3 old_Rev	5’ TCTCCAAACCAAGGTAAACACATA 3’	
